# Performance of point-of-care birth HIV testing in primary health care clinics: An observational cohort study

**DOI:** 10.1371/journal.pone.0198344

**Published:** 2018-06-18

**Authors:** Bindiya Meggi, Lara Vojnov, Nedio Mabunda, Adolfo Vubil, Alcina Zitha, Ocean Tobaiwa, Chishamiso Mudenyanga, Dadirayi Mutsaka, Timothy Bollinger, Osvaldo Loquiha, Trevor F. Peter, Ilesh V. Jani

**Affiliations:** 1 Instituto Nacional da Saúde, Maputo, Mozambique; 2 Clinton Health Access Initiative, Maputo, Mozambique; 3 Department of Mathematics and Informatics, Universidade Eduardo Mondlane, Maputo, Mozambique; University of Cape Town Faculty of Health Sciences, SOUTH AFRICA

## Abstract

**Background:**

Failure to timely diagnose HIV in infants is a major barrier for scaling-up paediatric antiretroviral treatment (ART). WHO recommends birth testing for earlier diagnosis and to improve test coverage, but current diagnosis takes 2–3 weeks to complete, thereby limiting the ability of care givers to provide follow-on care, especially in low-resource settings. We evaluated the benefit of implementing rapid diagnosis of HIV at birth in primary health care maternity wards in Mozambique.

**Methods and findings:**

Infants born to HIV-infected mothers delivering consecutively at eight primary health care clinics were tested within 24 hours of delivery using on-site POC (Alere q HIV1/2 Detect) and standard laboratory (Roche COBAS AmpliPrep/TaqMan HIV-1 qualitative assay v2.0) testing. Infants were also tested at 4–6 weeks of age with both assays. Of 2,350 HIV-exposed infants enrolled in this implementation research study, 33 tested HIV-positive at birth on both assays. Sensitivity and specificity of POC testing compared with laboratory testing at birth were 100% (95% CI 89·4–100·0) and 100% (95% CI 99·8–100·0), respectively. At 4–6 weeks of age, 61 infants were identified as HIV-positive; of these 29 (47·5%) had a positive test at birth. Testing at both birth and 4–6 weeks identified 71 HIV-positive infants compared with 61 infants by testing at 4–6 weeks alone, a 16% increase. Two infants tested positive at birth but tested HIV-negative during follow-up.

**Conclusions:**

Adding POC birth testing to the 4–6 week screen may increase access to HIV diagnosis and expedite ART initiation in primary health care settings within low resource settings. Guidance on appropriate confirmatory HIV testing algorithms for birth testing is needed.

## Introduction

Worldwide only 43% of infants infected with HIV have access to antiretroviral treatment (ART) [1]. Despite significant global efforts to expand access to paediatric treatment, coverage lags behind adult ART and may not reach the 90-90-90 goals by 2020 as set by UNAIDS [2]. Resolving this paediatric ART gap is a public health priority in high HIV burden countries, particularly as mortality is high within the first 2–3 months of life and 50% of untreated HIV-infected children die within the first two years of life [3,4].

Failure to diagnose HIV-infected infants soon after birth is one of the leading causes for low paediatric ART coverage. Fewer than 43% of HIV-exposed infants in low and middle-income countries are tested within two months of age [1].

This is often because early HIV infant diagnosis (EID) tests are only provided at the 4–6 weeks post-natal visit, and not when infants present for care in other settings [5,6]. To address this, WHO recommends a number of strategies for identifying HIV-infected infants at other entry points of the health system, including testing at birth [7].

Birth testing enables early diagnosis of *in utero* infected infants and may be an important addition to routine 4–6 week screening, especially in settings where post-natal retention is low or when mothers seek follow-on care at other health facilities [6,8–10]. However, in many resource-limited countries, mothers remain at maternity facilities for less than 24–36 hours after delivery and current EID approaches can take up to four weeks or longer to complete [11]. Point-of-care (POC) EID may enable rapid on-site birth testing in maternity facilities, and early treatment and prevention support before infants leave the facility. Previous studies have shown that POC and near POC technologies used for EID have comparable analytical performance with laboratory-based instruments [9,12–16]. Furthermore, HIV POC testing of infants at 4–6 weeks of age led to significantly reduced test turnaround times and increased ART initiation rates [17]. We therefore evaluated the feasibility, performance and diagnostic yield of rapid POC EID at birth within primary health care maternity wards in Mozambique.

## Methods

### Study design and participants

Participants in this prospective, observational study were tested using both POC and laboratory-based nucleic acid tests for HIV EID. Patient enrolment and POC testing were conducted at eight public primary health care clinics located in Maputo City, Beira City and Maputo Province, namely 1° de Maio, Polana Caniço, Albazine, José Macamo, Mavalane, Munhava, Ponta Gêa and Xinavane. Participants eligible for this study were infants born to HIV-positive mothers aged over 18 years (regardless of their ART history) at the maternity wards of the participating health facilities, whose mothers or guardians provided informed consent, and who could be tested between four and 24 hours of age. Infants excluded from the study were those older than 24 hours of age, those not born at the participating health facilities, and those with serious medical conditions, delivery complications, born through Caesarean section, or born to mothers with mental illness. Demographic, clinical, and test data for study participants were collected using study specific forms, and patient identifying information was coded in order to maintain confidentiality.

### Test methods

HIV-exposed infants were tested at maternity wards by trained nurses using the Alere q HIV-1/2 Detect system (Alere Inc, Waltham, Massachusetts, USA) within 24 hours of birth. This test detects total HIV-1/2 RNA in whole blood specimens and was performed just after specimen collection. Dried blood spot specimens (Whatman 903, GE Healthcare Biosciences, Pittsburgh, PA, USA) were simultaneously drawn from heel or toe pricks, and transferred within one week for blinded testing at central reference laboratories using the Roche CAP/CTM 96 HIV-1 Qualitative Test v2 (Roche Molecular Diagnostics, Branchburg NJ, USA). This test detects HIV-1 total nucleic acid (DNA and RNA) when used on whole blood samples. The reference laboratories routinely participated in and passed external quality assessment programs (provided by the Centers for Disease Control and Prevention, Atlanta, USA) prior to and during the study.

Laboratory and POC birth test results were not used for patient diagnosis as they were not part of routine care. The cohort of infants tested at birth was followed-up and tested again with both laboratory and POC assays for the routine EID screen at 4–6 weeks. After birth testing, participants were actively followed-up to undergo the 4-6-weeks EID screen via telephone call reminders by trained study counselors. Infants who missed the 4–6 week visit at study sites were traced through the National Early Infant Diagnosis Database and their laboratory EID test result was used for the study. Only 4–6 week laboratory EID results were provided to study participants and clinicians, per national guidelines. All at birth HIV positive infants were traced in order to get a fast track EID test at 4 weeks and start treatment as soon as possible after that.

### Analysis

The sensitivity and specificity of POC EID, as well as the positive and negative predictive values were estimated using laboratory EID as reference. Only valid results (positive or negative) were used for these calculations. Agreement between the POC EID and laboratory EID tests was assessed using the overall agreement measure and Cohen’s Kappa. Pearson’s chi-square test was employed to evaluate whether the positivity rate was associated with the demographic characteristics of patients. Inference was based on 95% confidence intervals for estimates of accuracy and agreement measures [18].

This study was approved by Mozambique’s National Health Bioethics Committee. Parents or legal guardians of the HIV-exposed infants were invited to participate in the study and were provided with a detailed study information sheet. A signed informed consent form was obtained for each parent or guardian that agreed to their infant’s participation in the study. Informed consent was sought at least four hours after delivery.

## Results

**Fig 1:**
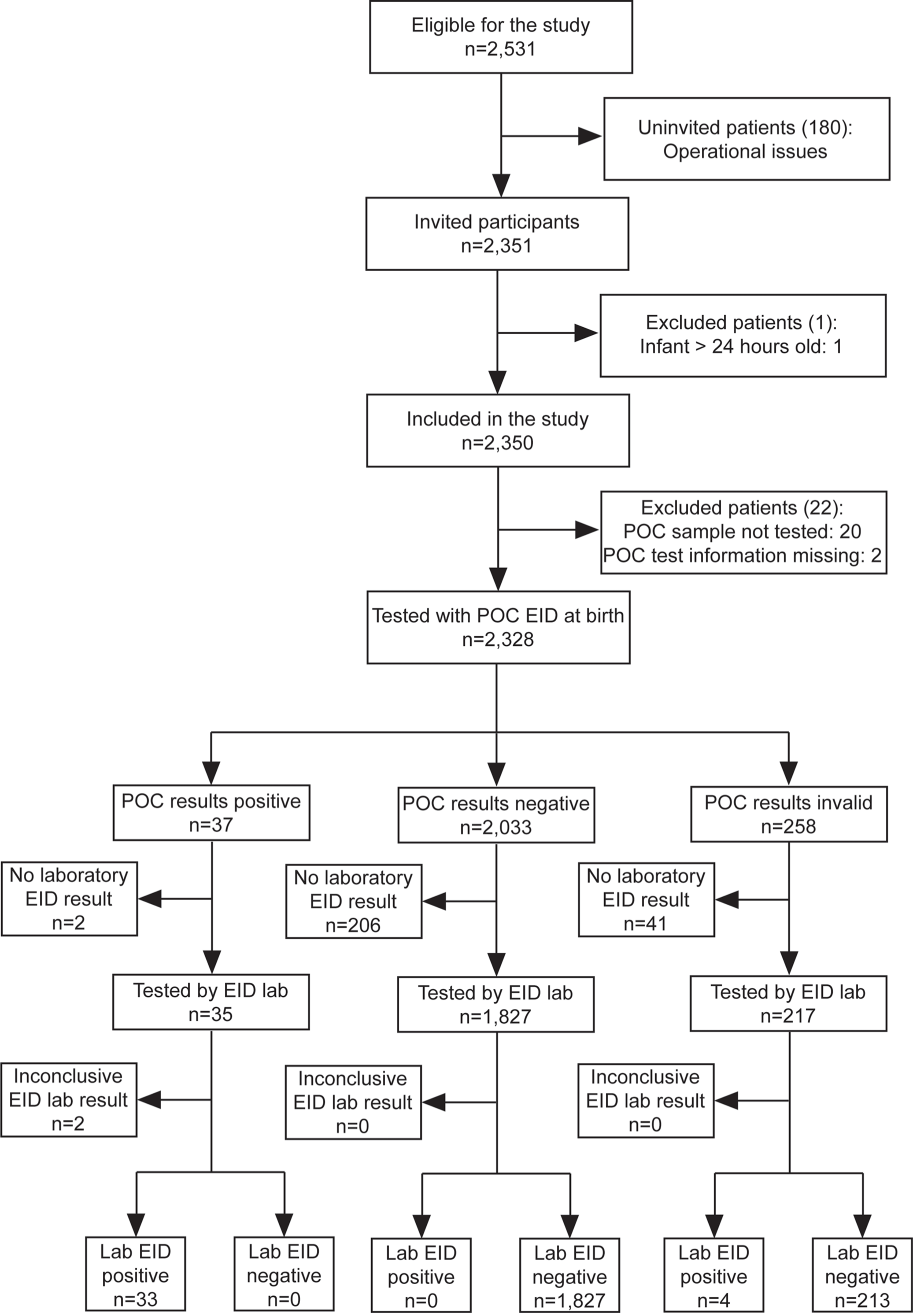
Flow diagram of participants at birth. POC = Point of Care, EID = Early Infant Diagnosis, Lab = laboratory test, POC Invalid = reported testing error, No Lab EID = poor quality or lost samples.

A total of 2,350 infants were enrolled in the study from November 2014 to July 2016 (Fig 1). Approximately 50% of infants were female (Table 1). The median age of infants tested at birth was one day (IQR: 0–1), while the median age of infants tested for routine 4–6 week EID was 31 days (IQR: 30–34). The majority (84%) of mothers were receiving ART (Option B+) and 60% of infants received nevirapine prophylaxis. There was no association between HIV status and gender, median age and maternal or infant treatment regimens, except for maternal treatment regimen at birth (p = 0.007). Twenty-two participants were excluded from the study, 20 due to the patient sample not being tested using the POC assay and two due to unrecorded POC test results. Of the remaining infants, 2,328 had a specimen collected for EID testing at birth and 2,077 were successfully screened at birth using both EID tests. POC testing yielded 258 invalid results, while 249 infants did not obtain a laboratory EID test result due to poor quality (79.9%; n = 199) or lost specimens (20.1%; n = 50).

**Table 1:** Study population characteristics at birth and follow-up HIV testing at primary health clinics in Mozambique.

**Birth laboratory Test**									
		**Total**		**Negative**		**Positive**		**Invalid**	
		**n**	**%**	**n**	**%**	**n**	**%**	**n**	**%**
Total		2350	100.0%	2051	87.3%	37	1.6%	262	11.1%
Sex *(p = 0.064)^†^*	Female	1185	50.4%	1024	86.4%	24	2.0%	137	11.6%
	Male	1141	48.6%	1005	88.1%	12	1.1%	124	10.9%
	Data not available	24	1.0%	22	91.7%	1	4.2%	1	4.2%
Mother regimen *(p = 0.007)^†^*	None	13	0.6%	10	76.9%	2	15.4%	1	7.7%
	Option A	11	0.5%	10	90.9%	1	9.1%	0	0.0%
	ART	1981	84.3%	1733	87.5%	29	1.5%	219	11.1%
	Data not available	345	14.7%	298	86.4%	5	1.4%	42	12.2%
**Routine laboratory Early Infant Diagnosis Test**									
Total		2350	100%	1501	63.9%	59	2.5%	790	33.6%
Sex *(p = 0.894)^†^*	Female	1185	50.4%	746	63.0%	30	2.5%	409	34.5%
	Male	1141	48.6%	742	65.0%	28	2.5%	371	32.5%
	Data not available	24	1.0%	13	54.2%	1	4.2%	10	41.7%
Age *(p = 0.782)^‡^*	≤2 months	1597	68.0%	1326	83.0%	45	2.8%	226	14.2%
	2–6 months	130	5.5%	97	74.6%	9	6.9%	24	18.5%
	6–12 months	8	0.3%	5	62.5%	2	25.0%	1	12.5%
	Data not available	615	26.2%	73	11.9%	3	0.5%	539	87.6%
Mother regimen *(p = 0.058)^†^*	None	13	0.6%	9	69.2%	1	7.7%	3	23.1%
	Option A	11	0.5%	4	36.4%	2	18.2%	5	45.5%
	ART	1981	84.3%	1427	72.0%	54	2.7%	500	25.2%
	Data not available	345	14.7%	61	17.7%	2	0.6%	282	81.7%
Infant regimen *(p = 0.051)^†^*	None	9	0.4%	6	66.7%	2	22.2%	1	11.1%
	NVP	1442	61.4%	1220	84.6%	51	3.5%	171	11.9%
	AZT	24	1.0%	18	75.0%	0	0.0%	6	25.0%
	Data not available	875	37.2%	257	29.4%	6	0.7%	612	69.9%
Infant breastfeeding *(p = 0.326)^†^*	No	41	1.7%	26	63.4%	2	4.9%	13	31.7%
	Yes	1174	50.0%	974	83.0%	41	3.5%	159	13.5%
	Data not available	1135	48.3%	501	44.1%	16	1.4%	618	54.4%

Option A = WHO recommended prophylaxis which includes: ante-partum AZT starting as early as 14 weeks gestation; intra-partum single-dose NVP and first dose of AZT/3TC at onset of labour; post-partum daily AZT/3TC for 7 days. NVP = Nevirapine. AZT = Zidovudine. ART = antiretroviral therapy. Invalid = Results not available or DBS sample rejected due to poor quality

† Fisher’s exact test p-values

‡ Non-parametric test for independent-samples medians p-value

The sensitivity and specificity of POC birth EID compared with laboratory birth EID was 100% (95% CI: 89·4–100·0, n = 33) and 100% (95% CI: 99·8–100·0, n = 1,827), respectively (Table 2). The positive and negative predictive values and overall agreement of POC testing at birth were 100% compared to laboratory testing, and Cohen’s kappa was 1·000.

**Table 2:** Results of at birth point-of-care testing with the Alere q HIV-1/2 Detect system compared with reference birth laboratory testing using the Roche CAP/CTM Qualitative HIV-1 assay.

		At birth Point-of-Care Early Infant Diagnosis		
		Positive	Negative	Total
At birth Laboratory Early Infant Diagnosis	Positive	33	0	33
	Negative	0	1827	1827
	Total	33	1827	1860

Eleven percent of POC birth tests and 3·1% of POC tests at 4–6 weeks did not produce a valid result due to test failure, while the rejection rate due to poor sample quality of laboratory dried blood spot specimens collected at birth and at 4–6 weeks was 9·1% and 11·2%, respectively (Fig 1). Four infant birth samples that tested positive with the laboratory test produced invalid results on the POC assay, while two infant birth samples that tested positive with the POC assay gave invalid results on the laboratory test. Sixteen percent of patients with an invalid POC result had no laboratory test result; this proportion was higher than those observed in infants with positive (5.4%) and negative (10.1%) POC results, p-value = 0.202 and 0.014, respectively (Fig 1).

**Fig 2:**
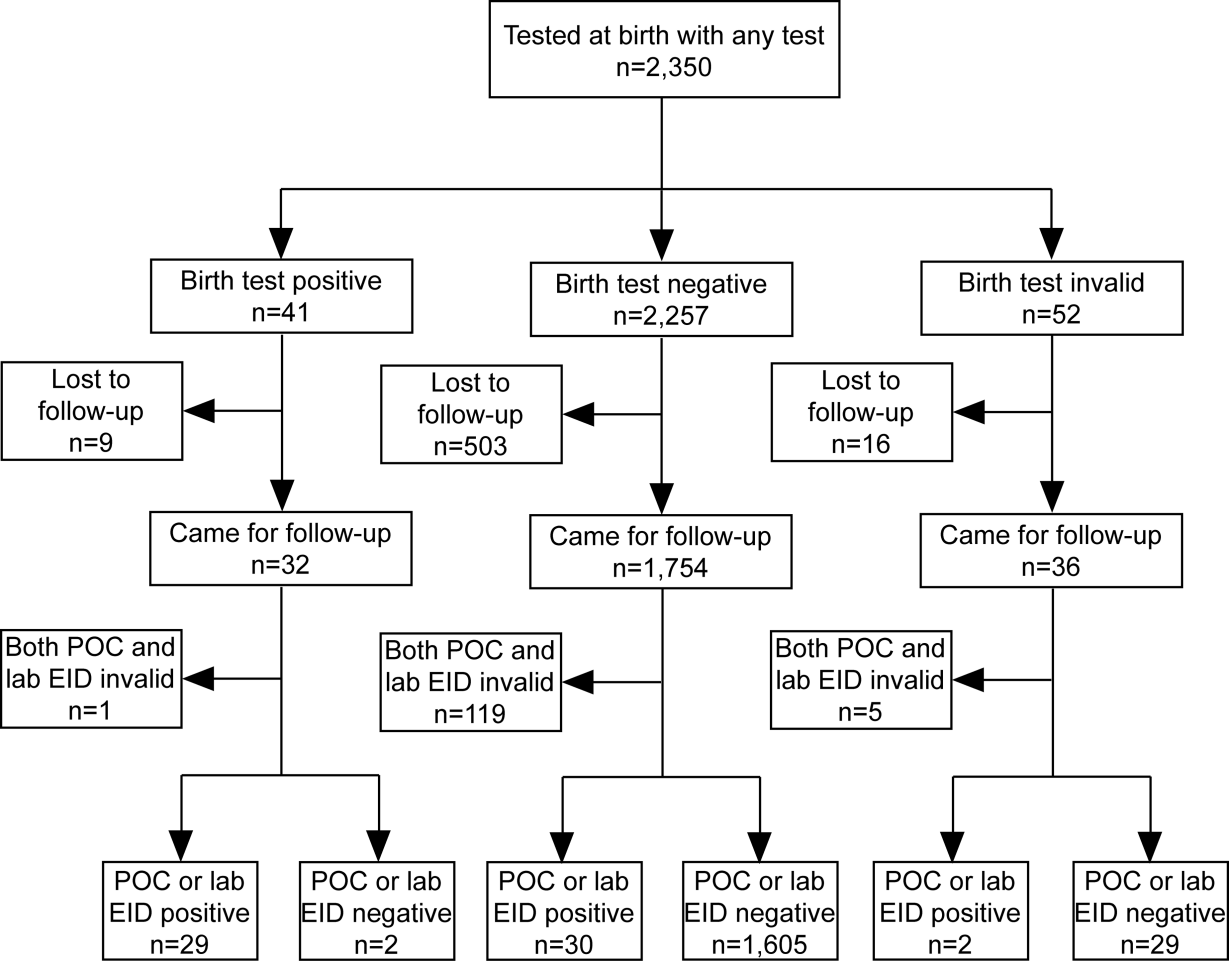
Flow diagram of participants at birth and at routine EID testing time point. Lost to follow up = patients tested at birth but who did not attend routine early infant diagnosis consultation, POC = Point of Care, EID = Early Infant Diagnosis, Lab = laboratory test.

Of the 61 infants identified as HIV-infected at 4–6 weeks, approximately half (n = 29) had detectable infections at birth. Furthermore, nine HIV-positive infants identified at birth were lost-to-follow-up prior to the 4–6 weeks EID test and one had an invalid result at 4–6 weeks EID test (Fig 2). Testing at both birth and 4–6 weeks identified a total of 71 positive infants compared to 61 positive infants identified with 4–6 weeks testing alone, resulting in a 16·4% (95% CI: 7·8%– 25·0%) increase in the number of HIV-infected infants diagnosed.

Two infants tested positive at birth with both POC and laboratory-based assays but tested negative at 4–6 weeks on at least one of the assays. Both infants tested positive when subsequently screened again at four months of age. Both these infants received nevirapine prophylaxis until 6 weeks (42 days) and both mothers were on ART. Additionally, two infants that tested positive on the POC assay at birth but that had an invalid laboratory test result, tested repeatedly negative after 4–6 weeks of age with POC and/or laboratory assays (Table 3).

**Table 3:** Patients with discordant test results between birth and after four weeks.

				At Birth Testing			≥4 Weeks			≥4 Weeks (repeat)		
	Mother Prophylaxis	Infant Prophylaxis	Infant Breast-feeding	POC	Lab	Age (Days)	POC	Lab	Age (Days)	POC	Lab	Age (days)
Patient 1	ART	NVP	Yes	Pos	Pos	1	Neg	DBS Rejected	31	Pos	Pos	142
Patient 2	ART	NVP	No	Pos	Pos	1	Not Processed	Neg	51	Pos	Pos	123
Patient 3	ART	NVP	Yes	Pos	Inv	1	Neg	DBS Rejected	32	Not Processed	Neg	127
Patient 4	None	NVP	Yes	Pos	Inv	1	Neg	DBS Rejected	29	Neg	Neg	63

POC = Point-of-Care test. LAB = laboratory test ART = antiretroviral therapy. NVP = Nevirapine. Pos = Positive. Neg = Negative. Inv = Invalid.

## Discussion

This study demonstrates that POC EID at birth is accurate and feasible when performed by nurses in low-resource primary health care clinics. Our results suggest that testing at both birth and 4–6 weeks may increase the proportion of HIV-infected infants diagnosed over the current practice of a test at 4–6 weeks alone. The use of POC tests may be essential to effectively implement birth testing, provide mother-infant pairs with immediate test results and initiate follow-on care before discharge from maternity wards. In many low-resource settings, standard laboratory EID takes up to four weeks or longer to return test results [19–21]. Even in settings with good testing infrastructure, laboratory-based EID can take at least three-four days to return results, by which time most newborns have been discharged from the maternity. Within the context of the low EID and paediatric ART access that presently limit achievement of the UNAIDS 90-90-90 goals by 2020, interventions that increase access to EID should have high priority. This also highlights the importance of strengthening POC diagnostic capacity and associated health systems to ensure on-site testing is feasible in low-resource settings [22].

Access to immediate HIV test results at birth may allow care providers to provide treatment earlier, as well as to focus on prevention strategies for uninfected infants. This may help reduce mortality in the first few weeks of life as well as prevent post-natal infections. This study demonstrated that testing at birth and at 4–6 weeks of age identified an additional 16% of HIV-infected infants than the 4–6 weeks test alone. Nine HIV-positive infants identified at birth did not return for the 4–6 weeks test, potentially due to mortality or other loss from care. Furthermore, nearly 50% of infants that tested HIV-positive at the 4–6 weeks time point were already positive at birth and thus could have initiated life-saving ART earlier. Birth testing may therefore increase coverage of EID and ensure earlier access to ART [23]. Birth testing has been shown to be cost-effective in South Africa [24]. Further studies are needed to confirm the generalizability of these observations as well as assess the cost-effectiveness and affordability of this approach in less resourced settings. Considering the relatively high costs per device of currently available POC EID technologies, thoughtful site selection processes may need to take affordability, access and disease burden into account until less costly devices or disposable technologies are available for widespread decentralization.

We identified two infants who tested positive for HIV infection with laboratory-based and/or POC EID nucleic acid tests at birth, but subsequently tested negative on these assays at two subsequent time points. Additionally, two infants had positive test results at birth and at approximately four months of age, but negative test results at 4–6 weeks. For these infants NVP prophylaxis may have induced negative PCR test results at 4–6 weeks, these becoming positive again after NVP was stopped. Similar observations have been made previously[25]; however, the cause is unclear. Further assessment of birth testing is therefore needed to determine whether the negative results in these cases are caused by low viraemia, exposure to antiretroviral drugs, or sample handling errors. These observations highlight the importance of confirmatory EID testing, though it may be difficult to confirm initially positive birth tests with a repeat test either at the same time (which still may not distinguish transient low viraemia from actual infection) or after ART has started if viraemia is suppressed. These potential difficulties with birth testing need to be resolved before its widespread use and additional evidence and guidance on confirmatory testing algorithms are needed to inform routine birth EID practices.

In this study, point of care EID testing on newborns was associated with an 11.1% test failure rate, a rate substantially higher than the 3.1% observed at 4–6 weeks. The difficulty of collecting blood samples from newborns may have been the cause for these differences. The rates observed in our study were similar to those seen in other studies, where rates ranged from 2–9% and were higher among younger infant [9,13,15]. Similarly, 9.8% of dried blood spot specimens from infants at birth and at 4–6 weeks of age failed to meet minimum quality standards for laboratory-based EID testing. A greater proportion of patients with an invalid POC result had no laboratory test result, when compared to infants with either a positive or negative POC results. This difference was statistically significant between those with invalid POC result when compared with those with negative POC result. The lack of statistically significance for patients with positive POC results may be due to the relatively small numbers in this group. We think that these observations may be related to the poor quality specimen collection in a group of newborns. Furthermore, because birth and point-of-care testing were not part of national policies and guidelines at the time of the study, those test results were not used for patient management. Thus, those infants with positive results at birth or using the POC technology did not undergo confirmatory testing. Confirmatory testing was, however, conducted for infants tested at 4–6 weeks using laboratory-based testing as per national guidelines. Once included in national guidelines and implemented into routine patient management practices, confirmatory testing at birth and when using point-of-care tests will continue to be critical as will understanding the impact of repeat testing when errors are encountered.

This study had several limitations. First, the sample size of HIV-positive infants was small because of Option B+ implementation. This positive development limits the feasibility of large sample size studies on HIV diagnosis among infants[26]; however, our data are strongly indicative that POC testing infants at birth may result in increased case-finding. Second, mothers at birth screening were encouraged and compensated to return for the routine EID test; therefore, the retention between birth testing and the 4–6 week EID screen observed here may be higher than in routine clinical settings. Additionally, this study was not designed to investigate transiently positive or negative test results; further studies are needed to understand the reasons for and extent of this phenomenon. This study was also not designed to investigate other critical outcomes such as treatment initiation, retention on treatment, viral suppression, morbidity or mortality. Finally, this study did not attempt to report on key feasibility, operational, and implementation aspects of POC and/or birth testing introduction, such as training, site selection, service and maintenance, instrument breakdown, waste management and affordability. Future studies or reports would add programmatic value.

Due to the lack of national policy and access to appropriate treatment for newborns at the time of the study, infants identified as HIV-positive at birth had to wait until the appropriate age for retesting according to national guidelines. Because of this, we were unable to investigate the impact and implications of testing at birth and immediate ART initiation. Treating newborns will likely imply a set of challenges requiring research and better understanding, including stigma and disclosure best practices, availability of suitable drug formulations, appropriate treatment dosing, effective linkage interventions and retention.

In conclusion, we have demonstrated that POC HIV EID at birth is feasible and accurate when conducted by nurses in primary health care clinics in low-resource settings, and may be an important tool to expand access to birth testing. We also highlighted that birth testing in combination with routine 4–6 weeks screening may increase access to EID. However, implementing POC EID at birth will need confirmatory testing of positives and supportive health systems to ensure reliable testing and retention of infants after birth[8,9,23]. Nevertheless, POC testing may improve opportunities for newborns to access ART especially in decentralised and task-shifted low-resource settings.

## Supporting information

S1 File Study data set. (XLS)
